# A problem with problem solving: motivational traits, but not cognition, predict success on novel operant foraging tasks

**DOI:** 10.1016/j.anbehav.2016.02.006

**Published:** 2016-04

**Authors:** Jayden O. van Horik, Joah R. Madden

**Affiliations:** Centre for Research in Animal Behaviour, Psychology, University of Exeter, Exeter, U.K.

**Keywords:** cognition, foraging innovations, *Phasianus colchicus*, pheasants, problem solving

## Abstract

Rates of innovative foraging behaviours and success on problem-solving tasks are often used to assay differences in cognition, both within and across species. Yet the cognitive features of some problem-solving tasks can be unclear. As such, explanations that attribute cognitive mechanisms to individual variation in problem-solving performance have revealed conflicting results. We investigated individual consistency in problem-solving performances in captive-reared pheasant chicks, *Phasianus colchicus*, and addressed whether success depends on cognitive processes, such as trial-and-error associative learning, or whether performances may be driven solely via noncognitive motivational mechanisms, revealed through subjects' willingness to approach, engage with and persist in their interactions with an apparatus, or via physiological traits such as body condition. While subjects' participation and success were consistent within the same problems and across similar tasks, their performances were inconsistent across different types of task. Moreover, subjects' latencies to approach each test apparatus and their attempts to access the reward were not repeatable across trials. Successful individuals did not improve their performances with experience, nor were they consistent in their techniques in repeated presentations of a task. However, individuals that were highly motivated to enter the experimental chamber were more likely to participate. Successful individuals were also faster to approach each test apparatus and more persistent in their attempts to solve the tasks than unsuccessful individuals. Our findings therefore suggest that individual differences in problem-solving success can arise from inherent motivational differences alone and hence be achieved without inferring more complex cognitive processes.

## Highlights

• Problem-solving performance was assessed in artificially reared pheasant chicks.
• Performance was consistent within same/similar tasks but not across different tasks.
• Successful solvers did not improve performance with trial and error experience.
• Differences between successful and unsuccessful birds were mediated by motivation.
• Success at problem solving may not reveal cognitive differences between individuals.

Anecdotal reports of innovative foraging behaviours have been found to correlate positively with relative brain size among birds and primates, and have therefore been considered to be associated with cognitive evolution (Lefebvre et al., 2004, Lefebvre et al., 1997, Overington et al., 2009, Reader and Laland, 2002). A more structured approach used to investigate the relationship between innovative behaviours and cognition is to present subjects with novel, but ecologically relevant, operant foraging problems, yet the mechanisms that underlie success on problem-solving tasks remain unclear and require further investigation (Griffin and Guez, 2014, Thornton and Samson, 2012). Problem-solving tasks often adopt extractive foraging paradigms, where success generates an immediate food reward (Cole, Cram, & Quinn, 2011), but may also exploit other motivations which may be equally rewarding, such as access to begging offspring (Cauchard, Boogert, Lefebvre, Dubois, & Doligez, 2013) or mate attraction (Keagy, Savard, & Borgia, 2009). Individuals are then classified as either successful or unsuccessful, depending on whether they solve the problem as posed by the experimenter, or their performances may be differentiated based on the speed or number of errors required to solve a particular task. Consequently, successful individuals are considered to possess enhanced cognitive capacities over those that fail to solve the problem, or do so slower or with more errors (Cole and Quinn, 2012, Keagy et al., 2012). Yet, evidence to suggest that the general cognitive ability of individuals that succeed on operant problem-solving tasks is quantitatively different from that of unsuccessful individuals remains unclear (see Griffin & Guez, 2014). While successful individuals may improve their performance with experience on operant foraging problems (Benson-Amram & Holekamp, 2012), it is not clear whether successful problem solvers are consistently successful across problems, assayed through different cognitive domains. Although individuals that successfully learn to solve operant problems in social groups show greater capacities for learning asocial operant problems (Boogert, Reader, Hoppitt, & Laland, 2008) and innovative behaviours correlate positively with social learning (Bouchard, Goodyer, & Lefebvre, 2007), only a few studies have investigated whether successful problem solvers outperform unsuccessful problem solvers at learning other, more conventional psychometric problems, such as associative learning paradigms. Isden, Panayi, Dingle, and Madden (2013) found that problem solving clustered with the successful learning of several different types of tasks. Griffin, Guez, Lermite, and Patience (2013) also found that problem-solving latencies correlated positively with latencies to learn a colour discrimination problem. Yet, Tebbich, Sterelny, and Teschke (2010) found that the performances of tool-using woodpecker finches, *Camarhynchus pallida*, and nontool-using tree finches, *Camarhynchus parvulus*, on an operant foraging problem did not correspond with their performances on a colour discrimination problem.

Investigations into the mechanisms that underlie successful problem-solving performances have instead revealed traits not typically considered to be associated with cognition, including neophobia, age, persistence, sex and prior experience (see Griffin & Guez, 2014 for a comprehensive review). Object neophobia varies both within and between species and is likely to play an important role in problem-solving performance (Greenberg, 2003). Yet, support for the direction of this relationship is mixed and can also be closely related to an individual's age or social rank (see Griffin & Guez, 2014). Persistence of interactions with a test apparatus have also been considered important for success as it may result in a greater diversity of manipulations, and hence likelihood that a solution is achieved; yet success may also be driven by chance and require no causal understanding of the problem (Benson-Amram and Holekamp, 2012, Griffin and Guez, 2014, Thornton and Samson, 2012). Problem-solving performance may also be influenced by sex; yet again support for the direction of this relationship is mixed (Cole et al., 2011, Reader and Laland, 2001, Thornton and Samson, 2012). Finally, problem-solving performance may depend on prior life experiences including access to components used in the tasks in a different context, or interactions with human artefacts in general (van de Waal & Bshary, 2010). Unfortunately, in most cases, such prior experience is either unknown or not standardized. Consequently, these noncognitive factors may obscure explanations for individual differences in problem-solving performance, and as such need to be controlled or accounted for before cognitive explanations can be invoked.

A final hindrance to interpreting results from problem-solving tasks is that consistently few individuals within a population interact with the apparatus, and, of these, the majority fail to access the reward. For instance, rates of success as low as 15% of 62 hyaenas, *Crocuta crocuta* (Benson-Amram & Holekamp, 2012), 32% of 53 wild vervet monkeys, *Chlorocebus pygerythrus*, and 7% of 30 vervets from groups that had minimal exposure to humans (van de Waal & Bshary, 2010), 10% of 135 meerkats, *Suricata suricatta* (Thornton & Samson, 2012) and 14% of 236 great tits, *Parus major*, and blue tits, *Cyanistes caeruleus* (Morand-Ferron, Cole, Rawles, & Quinn, 2011) have been reported. While low success rates are consistent with the idea that innovations are rare events in populations, it remains important to demonstrate that this preponderance of failure is due to an individual's cognitive inability, rather than other, noncognitive mechanisms that mediate propensities to interact with operant foraging problems.

We addressed the above issues by assessing whether individual differences in problem-solving success involve cognitive mechanisms, such as learning and memory, or whether success can be better explained through noncognitive mechanisms. If problem-solving performances accurately indicate differences in individuals' cognitive abilities, then we expect successful individuals to be consistently successful not only across subsequent presentations of the same problem, but also across different types of problems. When presented with problems that can be solved by alternative methods, we expect successful individuals to adopt consistent techniques during subsequent exposures to the same problem, rather than switching to an untried solution, suggesting retention of previously learned information about task affordances. If success is mediated by learning, we predict that successful individuals will become more efficient, showing increased speed and decreased handling of the apparatus, across repeated trials of the same problem. Alternatively, individual differences in problem-solving success may be explained by other factors that do not require cognitive mechanisms. If so, then we expect that individuals with particular motivational characteristics, such as those that rapidly approach the apparatus and are highly persistent in their interactions with the apparatus, will outperform less motivated and less persistent individuals.

Pheasant chicks, *Phasianus colchicus*, offer an excellent, if unusual, system to robustly test problem-solving performance on a large scale with rigorous control of potential confounds. Pheasants are precocial and can be artificially reared, hence removing parental influences early in life. Rearing in captivity provides an opportunity to control diet, hunger and exposure to novel stimuli. Large numbers of pheasants can be hatched simultaneously, and therefore by presenting problems on the same day to multiple individuals, age effects can also be controlled. Individuals can be shaped with food rewards to enter test arenas and interact with test apparatuses under their own volition. Pheasants can be visually sexed and crucially, when young, they readily interact with novel test apparatuses (J.O. van Horik & J.R. Madden, personal observation).

## Methods

### Subjects and Procedures

Two hundred pheasant chicks were housed in groups of 50 in four replicated 2 m^2^ enclosures between 28 May and 29 July 2014. Subjects were purchased as day-old chicks from a commercial game breeder and individually marked using numbered wing tags. Birds were fed commercial pheasant feed, supplemented with wild bird seed (ca. 5%) and supplied with water ad libitum. All birds were tested with a battery of psychometric tests (including those detailed in this study) from 10 days old, with equal exposure to all tasks. Each of the four enclosures contained one experimental chamber (75 cm^2^), in which subjects were individually tested while visually isolated from other test subjects. During each test session, each experimental chamber was monitored by an observer, who allowed the test subject to voluntarily enter the experimental arena and then recorded its behaviours on a given task. Subjects were presented with one trial per day and each task was presented twice over consecutive days to determine whether their performance improved with experience. Subjects participated in a total of six test sessions and received each task in the following fixed order to standardize carryover effects: Flip-Top, Flip-Cup, Petri-Dish. Only one task was presented per session. Subjects were deprived of access to their normal food supply (but not water) immediately prior to testing, for up to 2 h, although discarded food items remained in the housing arena. To mitigate any effects of social isolation on the test subject, two companion birds, which did not participate in any test procedures, were placed adjacent to the test arena, separated from the focal bird by a mesh barrier. Morphometrics (mass, tarsus length) were taken and bird sex confirmed by plumage features at 8 weeks old prior to release.

### Problem-Solving Apparatuses

**Figure 1:**
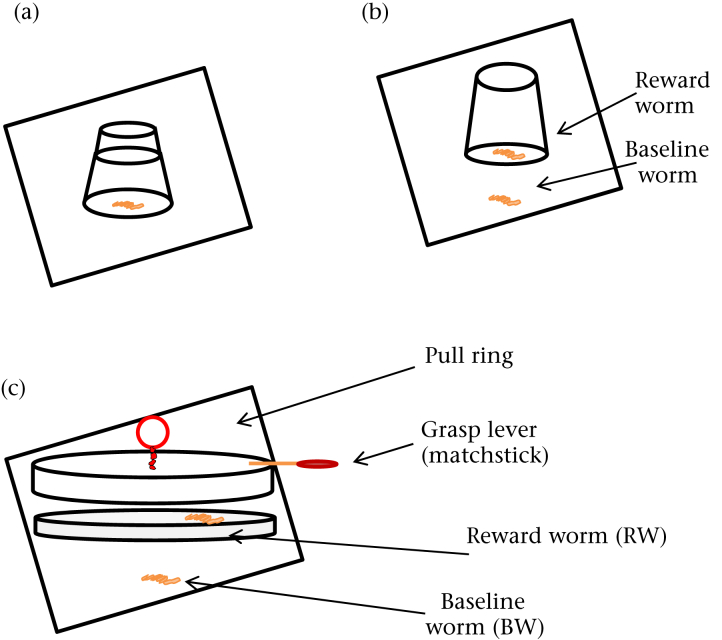
Schematics for each problem-solving apparatus: (a) Flip-Top problem which required subjects to flip off the top of a transparent shot glass to access a reward worm; (b) Flip-Cup problem which required subjects to flip over the entire shot glass to access a reward worm; (c) Petri-Dish problem which required subjects to remove the lid by either pulling at a ring or grasping at a lever to access the reward worm.

All chicks were given two similar extractive foraging tasks (Flip-Top and Flip-Cup) based on variants of the same apparatus but each requiring different solutions when they were 13 days old (Fig. 1a,b). Owing to prerelease time constraints, a randomly selected subset of 100 birds were then given another extractive foraging task (Petri-Dish; Fig. 1c) using a different apparatus, which required a different solution to the similar tasks, when they were 62 days old. The apparatus for each task was presented on a white Perspex baseplate (25 × 25 cm) with a live mealworm placed 4 cm from the test apparatus in the direction of the entry door to the test chamber. The time to acquire this worm (the baseline worm: BW) provided a standard measure of propensity to approach the apparatus, suggesting that subjects' motivation to interact with the apparatus was driven by the prospects of obtaining a food reward. In each task, the test apparatus contained a second live mealworm (the reward worm: RW), which could be accessed if the bird solved the problem. We recorded whether a subject accessed the RW, the time to acquire the RW and the number of pecks directed towards the apparatus as a measure of persistence. The order that each subject individually entered the experimental chamber was recorded as their test order (TO). Hence, as there were four replicated test arenas, each containing 50 subjects, test subjects were each allocated a TO score between 1 and 50 for each of the six trials. These scores were then used to assess participation and success for each trial. A low TO score was considered to reflect a stronger motivation to enter the test chamber. Subjects that acquired the BW were considered to have participated in the experiment, even if they did not subsequently acquire the RW. Subjects that acquired the RW were considered successful. Trials ceased when subjects successfully acquired the RW, or after 120 s for unsuccessful individuals.

#### Similar tasks

##### Flip-Top

An upturned transparent plastic shot glass (40 mm high × 20 mm diameter; Fig. 1a), containing the RW was positioned in the centre of the baseplate. The base of the shot glass was fixed by two wire loops to the baseplate so that it could not be knocked over. The top of the upturned shot glass comprised a lid that could be removed to access the RW. Subjects were required to peck the lid off the shot glass and then place their head inside the shot glass to retrieve the RW.

##### Flip-Cup

In this problem the shot glass (as above) was hinged to the baseplate by a single wire loop, allowing the entire glass to be flipped over to access the RW (Fig. 1b).

#### Petri-Dish problem

A clear petri dish (90 mm diameter) was placed at the centre of the baseplate and covered by a transparent lid which could be removed by one of two methods: grasping at a matchstick (40 mm) glued to the side of the lid and acting as a lever or pulling a red wire loop (15 mm diameter) fixed to the lid centre.

#### Control tasks

We determined whether subjects' behaviours were motivated by the RW, i.e. whether problem solving could be described as goal directed. Individuals were presented with an identical apparatus as above with a BW, but lacking the RW. A random selection of subjects (*N* = 21) were selected to participate in a Flip-Cup Control trial. These subjects participated in the Control trial on the day after the test trials had ceased. Subjects presented with the Petri-Dish Control (*N* = 28) were randomly selected from those individuals that had no prior experience with the Petri-Dish problem. Subjects received only one Control trial to maintain their motivation to interact with the apparatus.

### Statistical Analysis

Binomial tests were used to compare participation (participate versus nonparticipate) and success (success versus fail) across trials for the Test and Control conditions. We used McNemar tests to determine whether the proportion of individuals that succeeded on the Flip-Cup Control condition differed from those that succeeded on the Flip-Top and Flip-Cup Test conditions. Yates-corrected chi-square tests were used to determine whether the proportion of successful individuals differed between the Control and Test conditions for the Petri-Dish task. Control trial performances were compared against subjects' performances on Trial 1 for each task. We used a one-way ANOVA to assess whether subjects' performances on the Petri-Dish Test were goal directed by comparing (1) the number of pecks made towards the Test and Control apparatuses and (2) whether the location pecks were directed towards the lid or peripheral components of the apparatus. Repeated measures ANOVAs were used to determine whether the performances of successful and unsuccessful individuals differed, and whether subjects became more efficient problem solvers across trials within a task. Hence, we measured (1) latencies to acquire the BW and (2) the number of pecks to acquire the RW. Planned, uncorrected, paired *t* tests were used to compare latencies to acquire the BW and number of pecks among successful and unsuccessful individuals across trials. To assess whether successful and unsuccessful individuals were consistent in their problem-solving performance we calculated repeatability of persistence, i.e. pecks, and BW acquisition latencies across Trials for each Task following Lessells and Boag (1987). Binomial tests, set at 0.5 (same versus different), were used to assess whether subjects used consistent techniques to solve repeated presentations of the same problem.

We used generalized linear models (GLMs) to assess individual consistency in participation and success across trials and tasks. Subjects' participation (acquisition of the BW) and success (acquisition of the RW) were coded as binomial response variables and, as subjects were only presented with each task twice, *Z* values are reported rather than repeatability correlations. Owing to low rates of success and participation, we controlled for zero inflation in GLMs using the syntax (link=‘cloglog’) attached to the family. Generalized linear mixed models (GLMMs) were used to assess noncognitive aspects of problem-solving performance, with Trials nested within Tasks as explanatory factors and individuals included as random effects. The following factors were included in the analysis to address influences on participation or success: (1) latency to acquire a BW; (2) the number of pecks; (3) TO score, i.e. the order that subjects voluntarily entered the test arena; (4) body condition (mass/tarsus length^3^); and (5) sex. All GLMs and GLMMs were conducted in R version 3.2.2 (R Development Core Team, 2008) using the lme4 package (Bates, Maechler, Bolker, & Walker, 2015). All main effects and their two-way interactions were initially included in each model and we used stepwise reduction, based on *P* values, to determine best fitting minimal models. All tests were two tailed and differences between groups were considered significant when *P* < 0.05.

### Ethical Note

All work was approved by the University of Exeter Psychology Ethics Committee and conducted under Home Office licence PPL 30/3204. Birds were habituated to human observation from 1 day old. Shaping procedures, using mealworm rewards, were adopted to habituate subjects to the test arena. These procedures were considered to alleviate stress and encourage subjects' voluntary participation during testing. Birds could therefore choose whether or not to participate in tasks. There were no enforced aversive stimuli. To encourage participation in the tests, birds were removed from their normal food supply (but not water) for up to 2 h before testing while in the holding section. Birds that failed to engage with the task in 2 min were permitted to pass into a recovery area and their lack of participation recorded. Birds were reared at a lower density than that recommended by DEFRA's code of practice, thus reducing likely stress and competition between chicks.

## Results

### Are Participation and Success Goal Directed?

**Figure 2:**
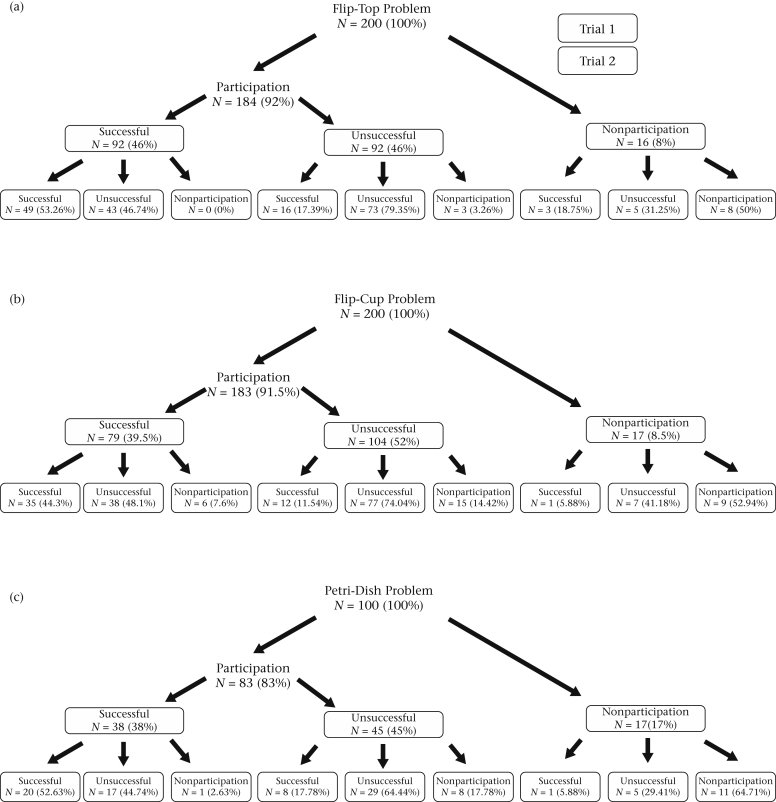
Performances of successful, unsuccessful and nonparticipating individuals across Trials 1 and 2 for each problem-solving task: (a) Flip-Top; (b) Flip-Cup; (c) Petri-Dish. Individuals that acquired the baseline worm (BW) were considered to have participated; individuals that failed to acquire the BW were considered nonparticipators. Individuals that acquired the reward worm (RW) were considered successful; those that failed were considered unsuccessful.

Subjects were significantly more likely to participate, i.e. acquire the BW, than not participate in all Test trails (binomial test: all *P* < 0.001). Hence, the majority of birds were motivated by the presence of the BW (Fig. 2). Conversely, less than half of our subjects successfully obtained the RW in any one task (Fig. 2). As such, subjects were significantly more likely to fail than succeed in all Test trials (binomial test: all *P* ≤ 0.02) except on Trial 1 of the Flip-Top problem, in which success did not differ significantly from chance (binomial test: *P* = 0.29). When presented with the Flip-Cup Control apparatus, 20 of 21 individuals (95%) obtained the BW (binomial test: *P* < 0.001). Similar findings were observed on Trial 1 of the Flip-Top (92%) and Flip-Cup (91.5%) tasks. The presence of the RW was also crucial in motivating individuals to solve the Flip-Top and Flip-Cup Tasks. Only two of the 20 individuals (10%) that took the BW also solved the Flip-Cup Control apparatus when no RW was present (binomial test: *P* < 0.001), compared to 92 of 184 individuals (50%) on the Flip-Top task and 79 of 183 individuals (43%) on the Flip-Cup task (for Trial 1) when the RW was present. As such, subjects were significantly more likely to succeed when presented with each rewarded Test apparatus than on the unrewarded Flip-Cup Control apparatus (McNemar test: Flip-Top: *P* < 0.001; Flip-Cup: *P* < 0.001). Although we attempted to avoid any selection bias by randomly assigning subjects to the Flip-Cup Control condition, their previous experience on the Flip-Top and Flip-Cup Test trials may have influenced their subsequent performance on the Control trial. However, all Control condition subjects retrieved the BW in at least two of the four Test trials (median = 4, range 2–4) and pecked at the Test apparatus in at least one trial (median = 2, range 1–4), suggesting that Control subjects were not simply avoiding each apparatus.

**Figure 3:**
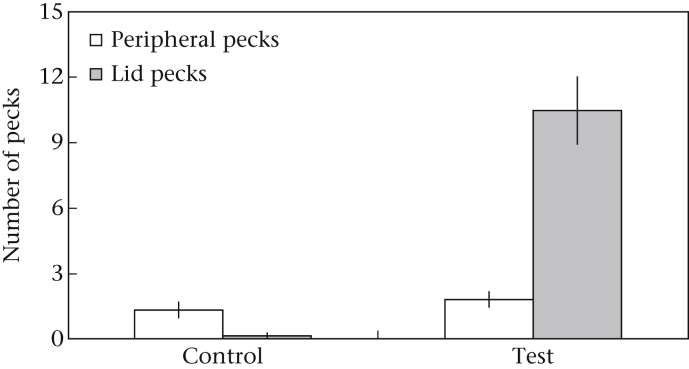
Mean ± SEM of number and location of pecks directed towards the unbaited Petri-Dish Control apparatus versus the Trial 1 performances of all birds on the baited Test apparatus. Pecks directed towards the apparatus lid are considered lid pecks, while pecks directed towards the matchstick or pull ring are considered peripheral pecks.

The RW was more critical in the Petri-Dish Control task with only 17 of the 28 individuals (61%) taking the BW (binomial test: *P* = 0.35), compared to 83% in Trial 1 of the Test condition (Fig. 2c). Of those 17 birds that acquired the BW in the Control task, 12 (70.6%) successfully removed the Petri-Dish lid and hence ‘solved’ the problem, even in the absence of the RW (binomial test: *P* = 0.24); compared to 38 of the 83 individuals (46%) in the Test trials. Consequently, subjects were significantly more likely to succeed (i.e. remove the lid) in the unbaited Petri-Dish Control condition than in the baited Test condition (chi-square test: χ^2^_2_ = 12.87, *P* < 0.001). Subjects in the Control condition, however, were more attentive to the peripheral components of the task, i.e. the matchstick and wire loop, which is likely to have facilitated their ‘success’. For instance, only one of the 12 successful birds (8%) pecked at the apparatus lid in the Control condition, while 68 of the 79 (86%) did so in the Test condition. The presence of the RW hence influenced the location and number of pecks that subjects directed towards the tasks. In the Petri-Dish task (the only task where the location of pecks was recorded), subjects pecked more frequently at the apparatus lid when the RW was present (Fig. 3; one-way ANOVA: *F*_1,127_ = 12.09, *P* = 0.001), and no differences in the number of pecks to peripheral components (match/loop) were found between the Test and Control tasks (Fig. 3; one-way ANOVA: *F*_1,127_ = 0.42, *P* = 0.52). Overall, these findings confirm that individuals were motivated by the presence of the RW in the Test conditions. As subjects that were presented with the Petri-Dish Control trial had no prior experience, and hence reward history, with the Petri-Dish Test conditions, the removal of the lid on this task may be driven by exploratory motivation.

### Do Individuals' Performances Improve with Experience?

**Figure 4:**
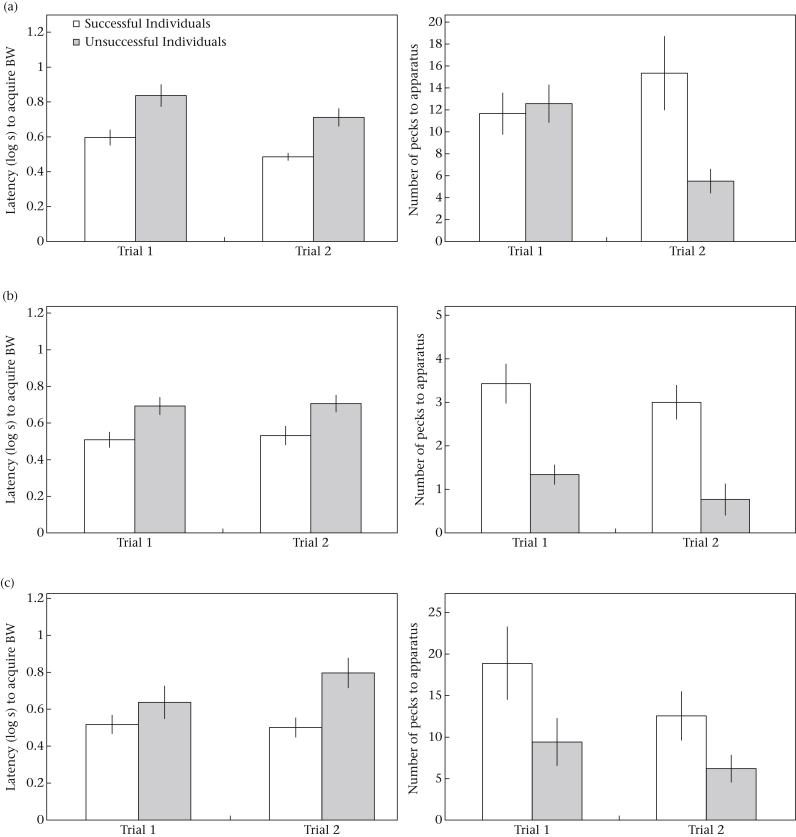
Mean ± SEM latencies to acquire a baseline worm (BW) positioned adjacent to each test apparatus and number of pecks directed towards each apparatus for individuals that were successful (white bins) and unsuccessful (grey bins) on both trials for all three problem-solving tasks: (a) Flip-Top; (b) Flip-Cup; (c) Petri-Dish.

Successful subjects did not become significantly faster to acquire the RW on the second presentation of each task (paired *t* test: Flip-Top problem (Trial 1 mean = 36.31 s ± 4.08 SEM; Trial 2 mean = 42.19 s ± 4.50 SEM): *t*_48_ = 1.17, *P* = 0.25; Flip-Cup problem (Trial 1 mean = 36.30 s ± 5.43 SEM; Trial 2 mean = 34.68 s ± 5.87 SEM): *t*_34_ = 0.62, *P* = 0.54; Petri-Dish problem (Trial 1 mean = 37.40 s ± 6.87 SEM; Trial 2 mean = 23.75 s ± 5.45 SEM): *t*_19_ = 1.84, *P* = 0.08), nor did they reduce the number of pecks required to solve a task over repeated trials (Fig. 4; paired *t* test: Flip-Top problem (Trial 1 mean = 11.66 ± 1.91 SEM; Trial 2 mean = 15.34 ± 3.37 SEM): *t*_48_ = 0.72, *P* = 0.47; Flip-Cup problem (Trial 1 mean = 3.43 ± 0.46 SEM; Trial 2 mean = 3.00 ± 0.40 SEM): *t*_34_ = 0.97, *P* = 0.34; Petri-Dish problem (Trial 1 mean = 18.90 ± 4.41 SEM; Trial 2 mean = 12.55 ± 2.97 SEM): *t*_19_ = 1.20, *P* = 0.25). Subjects acquired the BW faster on Trial 2 (mean = 11 s ± 1.20 SEM) than Trial 1 (mean = 16 s ± 1.24 SEM), of the first task that they faced (Fig. 4; Flip-top; repeated measures ANOVA: *F*_1,119_ = 8.05, *P* = 0.005). Yet no effect of trial on latencies to acquire the BW was observed for subsequent tasks (Fig. 4; repeated measures ANOVA: Flip-Cup: *F*_1,110_ = 0.14, *P* = 0.71; Petri-Dish: *F*_1,48_ = 1.21, *P* = 0.28).

### Does Previous Experience Correspond with Subsequent Performance?

#### Consistency in participation and success within tasks

Individuals were consistent, both in their likelihood of participating (acquiring the BW) and success (acquiring the RW), within repeated trials on a given task (Fig. 2). The likelihood of participating in the second trial of a task was best predicted by the individual participating in Trial 1 of that problem (GLMs: Flip-Top: *Z* = 4.60, *P* < 0.01; Flip-Cup: *Z* = 5.81, *P* < 0.01; Petri-Dish: *Z* = 4.15, *P* < 0.01). Similarly, the likelihood of succeeding in the second trial of a task was best predicted by their success in the first trial (GLMs: Flip-Top: *Z* = 4.72, *P* < 0.01; Flip-Cup: *Z* = 6.01, *P* < 0.01; Petri-Dish: *Z* = 4.01, *P* < 0.01). Success in Trial 2 of a task was not predicted by participation in Trial 1 (GLMs: all Tasks: *Z* < 1.66, *P* > 0.10), nor was participation in Trial 2 predicted by success in Trial 1 (GLMs: all Tasks: *Z* < 0.22, *P* > 0.09).

#### Consistency in participation and success across tasks

An individual's success on the Flip-Cup task could be accurately predicted by its success on the relatively similar Flip-Top task (GLMs: Trial 1: *Z* = −4.51, *P* < 0.01; Trial 2: *Z* = −3.67, *P* < 0.01). However, success in either of these tasks did not predict success in the dissimilar Petri-Dish task (GLMs: all *Z* < |0.86|; *P* > 0.34).

#### Consistency in problem-solving techniques

The Petri-Dish problem could only be solved, i.e. the lid removed, by one of two methods; either grasping the wire loop or levering the lid off with the attached matchstick. No additional methods of success were observed. Individuals that were successful in the Petri-Dish task in Trial 1 did not consistently use the same method in Trial 2 of the same task (binomial test: same *N* = 11, different *N* = 9, *P* = 0.82).

#### Repeatability of successful and unsuccessful individual performances

Successful and unsuccessful individuals showed low repeatability of BW acquisition latencies and pecks directed towards the test apparatus across trials for each task (Table 1).

### Can Performance be Predicted by Noncognitive Explanations?

#### Motivation to engage with the tasks

When testing commenced, those individuals that were among the first to voluntarily enter the test chamber (having a low TO score) were more likely to participate in each task (Table 2). Furthermore, individuals that rapidly approached the test apparatus (low BW latencies) were more likely to succeed (Table 2). Together, these findings suggest that motivational traits played an important role in determining whether or not individuals participated or succeeded in our problem-solving tasks.

#### Persistence

Subjects that pecked more frequently were more likely to succeed, i.e. acquire the RW, on our problem-solving tasks (Table 2). As such, successful individuals also pecked more frequently than unsuccessful individuals in all three problems (Fig. 4; repeated measures ANOVA: Flip-Top: *F*_1,119_ = 18.70, *P* < 0.001; Flip-Cup: *F*_1,110_ = 97.91, *P* < 0.001; Petri-Dish: *F*_1,48_ = 13.65, *P* = 0.001).

#### Sex, body condition and interactions between explanatory factors

**Figure 5:**
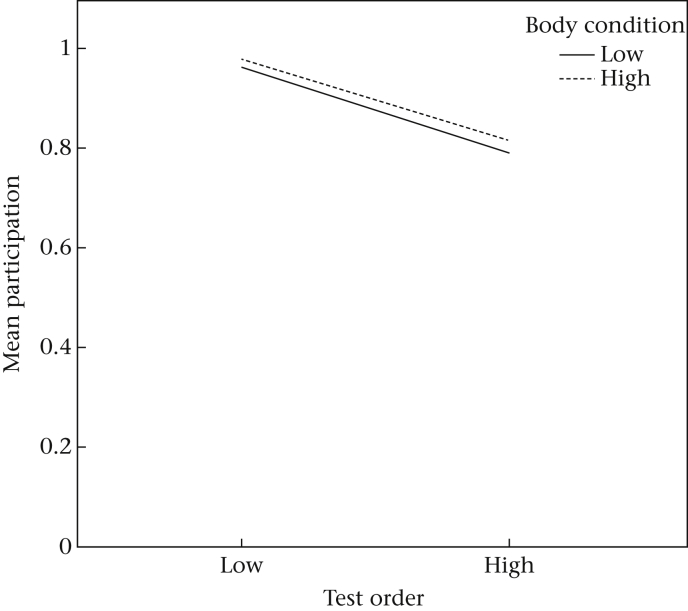
Influence of test order and body condition on participation (averaged across the six test trials). Test order represents the sequence that subjects voluntarily entered the test arena. Subjects were categorized into low or high body condition according to their respective below or above average scores.

We observed no significant effects of sex or body condition on participation or success (Table 2). A marginally significant TO*body condition interaction was, however, observed among participating individuals (Table 2). Individuals with a low TO score and high body condition index were more likely to participate (Fig. 5).

## Discussion

We presented pheasant chicks with three novel foraging tasks to assess whether their problem-solving performances were driven by cognitive or noncognitive, motivational, processes and whether these processes reliably predicted performance across repeated trials of the same problem, similar problems and different problems. When raised and tested in captivity under identical conditions, individuals showed consistency in whether or not they participated or succeeded, both within the same tasks and across similar tasks, but not across relatively different tasks. Yet latencies to approach each test apparatus and attempts to solve each problem, for successful and unsuccessful individuals, were not repeatable across trials. Successful individuals did not become more efficient problem solvers (by reducing latencies or peck numbers) across repeated presentations of the same problems, nor did they retain previously successful problem-solving techniques across subsequent trials, although the BW acquisition latencies of successful individuals improved marginally in one task, suggesting further experience may have facilitated learning. Overall, however, successful individuals showed no evidence of improvement through trial-and-error learning. Yet, an individual's likelihood of participation and success were predicted by noncognitive, motivational, traits. Our findings conform to those of previous studies, in which success on problem-solving tasks can be explained by differences in how individuals interact with novel foraging problems (Benson-Amram and Holekamp, 2012, Griffin et al., 2014, Thornton and Samson, 2012). Such differences do not arise from variation in age or previous experience, which we controlled for, and hence suggest that problem-solving success on some operant foraging tasks may be mediated by inherent individual differences in motivational traits alone.

### Are Participation and Success Goal Directed?

Few studies have previously addressed whether problem-solving performance is goal directed, as indicated by motivation dependent on a perceivable reward, and not simply an expression of exploring novelty in the individual's environment (but see Isden et al., 2013). The performances of subjects in our tasks relied on the presence of a visible reward (RW). As such, subjects were more likely to solve each task when the RW was present. Yet, some subjects' performances may also be explained by nongoal-oriented exploratory behaviours. A minority of individuals pecked at the control test apparatus and even successfully removed the lid, effectively ‘solving’ the task in the absence of the RW. However, subjects were generally more persistent and directed their pecks towards the RW rather than peripheral components when they were presented with a Test apparatus rather than a Control apparatus. Hence, task performance was primarily driven by a motivation to acquire the food reward and was thus considered goal oriented. However, the fact that some individuals interacted with the Control apparatus, even in the absence of a reward, suggests that interactions with novel objects, at least among a minority of individuals, may not be exclusively driven by a motivation to obtain a food reward. Future studies using problem-solving paradigms may benefit by clearly demonstrating that subjects' interactions with such tasks are driven by their motivation to access a reward, rather than success arising from novelty seeking and exploration of the test apparatus. Investigation into the relationships between exploratory behaviours and the discovery of hidden resources may also further our understanding of the mechanisms that underlie problem-solving success.

### Do Individuals' Performances Improve with Experience?

Successful pheasants failed to reduce their latencies or number of pecks to solve each task across trials, and did not retain previously successful techniques when repeatedly presented with the same task. Hence, we found no evidence that cognitive mechanisms associated with learning and memory facilitated the performance of successful pheasants. Latencies to acquire the RW on the second trial did, however, decrease marginally for successful individuals on the Petri-Dish problem, despite the relatively small sample size of individuals that participated in this problem. As such, the Petri-Dish problem may be the only task to reveal capacities for an increase in efficiency, as it requires grasping actions rather than persistent forward motion pecking. Hence, successful individuals may have significantly improved their performance with further experience. While findings from the current study contrast with those of previous studies, in which successful but not unsuccessful individuals improved their performance across subsequent presentations of the same problem (Benson-Amram and Holekamp, 2012, Bókony et al., 2013, Thornton and Samson, 2012), the performances of successful individuals in the current study do not appear to be driven by cognitive mechanisms. As such, success on these operant problem-solving tasks may be achieved irrespective of differences in learning ability. Future studies may therefore benefit by addressing whether their tasks reliably require cognitive processes such as learning and memory. As such, insight into the cognitive mechanisms that underlie problem-solving performance may be revealed by assessing whether an aptitude to solve operant problem-solving tasks also corresponds positively with performance on other classical psychometric paradigms involving learning and memory (Griffin et al., 2013a, Tebbich et al., 2010).

### Does Previous Experience Correspond with Subsequent Performance?

Individual participation and success were consistent across repeated trials of the same problem and within similar problems (Flip-Top and Flip-Cup tasks), but not across relatively different problems (between the Flip-Top/Flip-Cup and Petri-Dish tasks). Yet, successful and unsuccessful individuals showed poor repeatability in their BW acquisition latencies and pecks across trials on each task. Consistency in individual problem-solving performances across tasks is rarely assessed (but see Cole et al., 2011, Griffin and Diquelou, 2015, Griffin et al., 2013, Morand-Ferron et al., 2011, for good examples that successful individuals are consistently successful across trials, over time and on similar operant tasks). The differences between our Flip-Top and Flip-Cup tasks were not extreme, suggesting that similarities in subjects' participation and success between similar problems may have been facilitated by experience. In the similar tasks, two identical apparatuses (a clear upturned shot glass) were used, while in the different Petri-Dish task, the reward was still rendered inaccessible by a clear barrier. Despite this generality, we found that transfer of performance across relatively different tasks was poor. These findings may therefore be due to the different motor actions required to solve the dissimilar problems. As such, learning to peck over the cup on the Flip-Top and Flip-Cup tasks may not have assisted the performance of subjects on the Petri-Dish problem, which required subjects to grasp the matchstick or wire loop. Future work may therefore benefit from testing an individual's consistency in problem-solving performance across a variety of tasks of varying degrees of similarity in order to permit comment on generalized learning.

### Can Performance be Predicted by Noncognitive Explanations?

Individuals that were highly motivated to enter the test arena were also more likely to acquire the BW and hence participate in the tasks. Inherent motivational differences in individual propensities to interact with a test apparatus may therefore play an important role in problem-solving performance, and hence influence an individual's experience of reward outcomes. Successful individuals demonstrated lower latencies to acquire the BW than unsuccessful individuals. Differences in individual propensities to interact with novel objects may consequently lead to differences in life history experiences and hence what individuals understand about their surrounding environment (see Greenberg & Mettke-Hofmann, 2001). Such motivations may be mediated by object neophobia, although while highly neophobic individuals perform poorly in some studies (Benson-Amram and Holekamp, 2012, Benson-Amram et al., 2013, Boogert et al., 2006, Overington et al., 2011) they do not in others (Griffin and Diquelou, 2015, Morand-Ferron et al., 2011). While it remains difficult to differentiate whether subjects' latencies to acquire the BW were driven by differences in motivation or neophobia in the current study, we consider the latter explanation unlikely for the following reasons. Subjects' latencies to acquire the BW decreased significantly after the first trial of the first problem, but there was no significant effect of trial on subsequent problems. These findings suggest that subjects had habituated to the test environment after their first trial and hence that the subsequent performances of both successful and unsuccessful individuals were not influenced by differences in neophobia. Successful individuals, however, remained consistently faster at retrieving the BW than unsuccessful individuals, suggesting that differences in motivation to obtain a valuable food reward, rather than a general neophobia manifested by fear of the test apparatus, mediated their behaviours.

Successful pheasants were also more persistent, pecking more frequently at the apparatus, than unsuccessful individuals. Persistence of interactions has previously been considered an important component of problem-solving success (Morand-Ferron et al., 2011, Tebbich et al., 2010, Thornton and Samson, 2012), but may also impede success if individuals cannot inhibit making perseverative errors. In the current study, each problem-solving apparatus was designed so that the reward was clearly visible through a transparent Perspex barrier. Pecking directly towards the reward hindered access. Instead, subjects were required to manipulate peripheral components of each apparatus, and therefore inhibit inefficient prepotent actions directed towards the reward. Successful performance may also be due to enhanced capacities for inhibitory control, a cognitive trait associated with executive function (Amici et al., 2008, MacLean et al., 2014). However, we consider it unlikely that such cognitive attributes account for the successful performances we observed. We found that successful individuals did not reduce the number of pecks directed towards the apparatus across repeated trials, suggesting that they did not inhibit prepotent responses towards the reward (specifically avoiding pecking at a clear Perspex barrier) and that they failed to generalize similar rules across tasks. Instead we believe that, like hyaenas (Benson-Amram and Holekamp, 2012, Benson-Amram et al., 2013) and mynas, *Acridotheres tristis* (Griffin et al., 2014, Griffin and Diquelou, 2015), increased persistence and motor diversity may result in a greater variety of locations on the apparatus being manipulated, and hence an increased chance that successful manipulations were executed. Future work may therefore benefit from demonstrating that problem solving is not simply the result of effort, persistence and chance. As such, errors could be penalized, either empirically or in subsequent analyses, such that test animals can be designated as getting a problem wrong as well as getting it right, rather than simply failing to complete it (Thornton, Isden, & Madden, 2014). We found little evidence that sex or body condition in pheasants predicted their participation or problem-solving success. Yet we observed a marginally significant interaction where individuals that entered the test arena early (low TO) and had a high body condition index were more likely to participate.

Individual differences in problem-solving success in pheasants were unlikely to be due to different developmental experiences, which we standardized from hatching through controlled rearing conditions. We excluded opportunities for competition or social learning during testing. While we attempted to alleviate any stress caused by socially isolating the test subjects by including two companion birds in an annex of the test chamber, which allowed visual and acoustic interactions between the test and companion birds but restricted their physical contact, these procedures may also have some bearing on subjects' performance. Hence, we cannot exclude the explanation that individual variation in performance was influenced by differences in the responsiveness of the test birds to their close neighbours during foraging. Subsequent studies may therefore benefit by assessing whether individual variation in performance is consistent when subjects are also tested in social isolation. While most of our subjects were motivated to acquire the BW and attempted to solve each problem, we may have captured greater rates of success if we allowed subjects longer than 120 s in the test chamber. Indeed our subjects received less time to solve these problems than those in similar studies, yet subjects' performances in our study remained comparable to those previously reported. In contrast to previous studies, we also assessed performance among a relatively large number of individuals. Hence our capacity to test more individuals was offset by constraints on testing time.

To conclude, we assessed interindividual variation in problem-solving performance among pheasant chicks using three generic tasks, of which similar variants have previously been presented to a wide variety of taxa. While problem-solving assays have been considered relevant for the experimental evaluation of the mechanisms that underlie innovative behaviours (Griffin & Guez, 2014) observed among certain taxa that possess a relatively large brain size (Lefebvre et al., 1997), we consider that individual variation in problem-solving performance among pheasant chicks, may be mediated by inherent motivational differences alone. We do not consider successful performance to be driven by variation in cognitive abilities, such as learning, memory or executive control. Instead, successful individuals were better characterized by motivational traits, including low latencies to acquire the BW and enhanced persistence of object interactions. To illuminate the role that cognition plays in problem-solving success we suggest that future problem-solving assays control for or exclude noncognitive explanatory traits and include more classical measures of psychometric tasks to explicitly test cognitive processes of putative interest.

## Figures and Tables

**Table 1:** Repeatability of performances across trials for each task

Task	Successful	Unsuccessful
Flip-Top BW latency	*R*=0.02, *P*=0.02±0.14 SE	*R*=0.05, *P*=0.02±0.01 SE
Flip-Top pecks	*R*=−0.01, *P*=0.43±0.02 SE	*R*=0.12, *P*=0.001±0.05 SE
Flip-Cup BW latency	*R*=−0.02, *P*=0.74±0.17 SE	*R*=−0.01, *P*=0.84±0.08 SE
Flip-Cup pecks	*R*=−0.009, *P*=0.40±0.03 SE	*R*=0.17, *P*=0.001±0.01 SE
Petri-Dish BW latency	*R*=−0.05, *P*=0.82±0.01 SE	*R*=0.02, *P*=0.20±0.03 SE
Petri-Dish pecks	*R*=0.02, *P*=0.23±0.05 SE	*R*=0.006, *P*=0.28±0.03 SE

Repeatability (*R*) between Trial 1 and Trial 2, following Lessells and Boag (1987), for baseline worm (BW) acquisition latencies and number of pecks to the test apparatus for individuals that were either successful or unsuccessful on both trials for each problem-solving task.

**Table 2:** Factors predicting an individual's overall likelihood of participating or succeeding in each problem-solving task

	BW	P	TO	S	BC	TO*S	TO*BC	S*BC	BW*P	BW*S	BW*BC	P*S	P*BC	TO*BW	TO*P
Participation	n/a	n/a	−6.35; <0.001	0.99; 0.32	0.61; 0.54	0.21; 0.83	1.95; 0.05	0.99; 0.32	n/a	n/a	n/a	n/a	n/a	n/a	n/a
Success	−4.30; <0.001	4.35; <0.001	−1.35; 0.18	−0.53; 0.59	0.10; 0.92	1.14; 0.25	−1.43; 0.15	−1.05; 0.29	0.54; 0.59	0.12; 0.90	0.89; 0.37	−0.83; 0.41	−1.26; 0.21	1.17; 0.24	1.16; 0.24

Values reported are *Z* statistics and *P* values (significant *P* < 0.05) and are derived from best fit GLMMs. The following variables were included in each analysis: BW: baseline worm acquisition latency; P: pecks; TO: test order; S: sex; BC: body condition. All two-way interactions between the aforementioned variables are denoted by ‘*’. Subjects that failed to participate did not have P and BW scores and were therefore excluded from the analysis, denoted by ‘n/a’.
